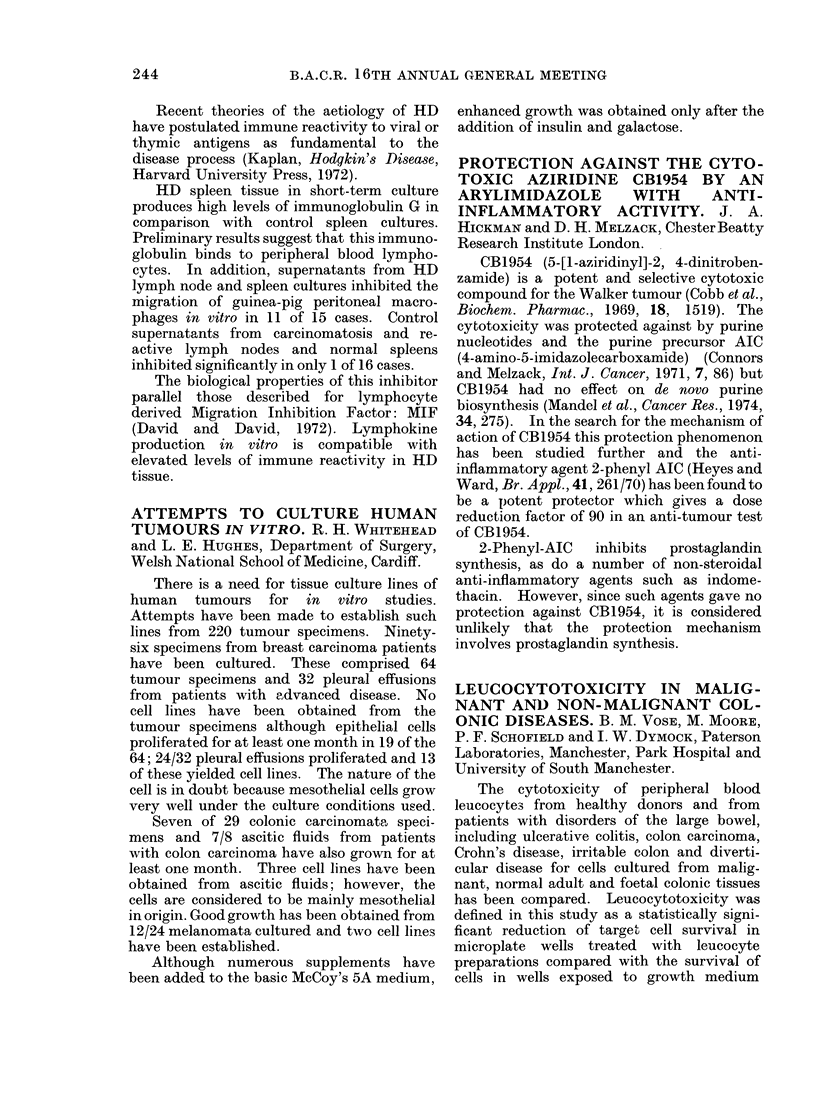# Proceedings: Protection against the cytotoxic aziridine CB1954 by an arylimidazole with anti-inflammatory activity.

**DOI:** 10.1038/bjc.1975.169

**Published:** 1975-08

**Authors:** J. A. Hickman, D. H. Melzack


**PROTECTION AGAINST THE CYTOTOXIC AZIRIDINE CB1954 BY AN ARYLIMIDAZOLE WITH ANTI-INFLAMMATORY ACTIVITY.** J. A. HICKMAN and D. H. MELZACK, Chester Beatty Research Institute London.

CB1954 (5-[1-aziridinyl]-2, 4-dinitrobenzamide) is a potent and selective cytotoxic compound for the Walker tumour (Cobb *et al.*, *Biochem. Pharmac.*, 1969, **18**, 1519). The cytotoxicity was protected against by purine nucleotides and the purine precursor AIC (4-amino-5-imidazolecarboxamide) (Connors and Melzack, *Int. J. Cancer*, 1971, **7**, 86) but CB1954 had no effect on *de novo* purine biosynthesis (Mandel *et al.*, *Cancer Res.*, 1974, **34**, 275). In the search for the mechanism of action of CB1954 this protection phenomenon has been studied further and the anti-inflammatory agent 2-phenyl AIC (Heyes and Ward, *Br. Appl.*, **41**, 261/70) has been found to be a potent protector which gives a dose reduction factor of 90 in an anti-tumour test of CB1954.

2-Phenyl-AIC inhibits prostaglandin synthesis, as do a number of non-steroidal anti-inflammatory agents such as indomethacin. However, since such agents gave no protection against CB1954, it is considered unlikely that the protection mechanism involves prostaglandin synthesis.